# The Role of Pancreatic Enzyme Insufficiency in the Etiology of Functional Dyspepsia Resistant to Standard Treatment

**DOI:** 10.5152/tjg.2025.255322

**Published:** 2025-10-31

**Authors:** Fatih Kemik

**Affiliations:** 1 Department of Internal Medicine, İstanbul University İstanbul Medical School, İstanbul, Türkiye

Dear Editor,

We would like to thank the correspondent for their thoughtful comments on our article entitled “The Role of Pancreatic Enzyme Insufficiency in the Etiology of Functional Dyspepsia Resistant to Standard Treatment.” We appreciate the opportunity to further clarify several aspects of our methodology and findings.

We acknowledge that the relatively small cohort size represents a limitation of our study. As stated in the discussion, this was the largest cohort-based analysis on this topic in our region; nevertheless, we agree that larger, multicenter, population-based studies are needed. Regarding statistical testing, we prioritized a descriptive and exploratory approach to generate hypotheses.^1^ While adjustments for multiplicity were not applied, our findings are consistent with previous reports, and we believe they still provide useful clinical signals warranting further investigation.

The concern regarding the inclusion of diabetic patients is well taken. Indeed, exocrine pancreatic insufficiency (EPI) is more common in patients with diabetes.^2^^,^^3^ However, this reflects real-world clinical practice: patients with both dyspeptic symptoms and diabetes are frequently encountered, and the differentiation between functional dyspepsia (FD) and diabetes-associated EPI is challenging. For this reason, we performed subgroup analyses and demonstrated that the prevalence of EPI was significantly higher in FD patients with DM (diabetes mellitus) compared to those without DM. Rather than confounding, we consider this an important clinical message that clinicians should maintain heightened awareness of possible EPI in dyspeptic patients with diabetes.

We agree that the Rome IV criteria classify patients with active *Helicobacter pylori* infection as “Hp-associated dyspepsia” rather than FD.^4^^,^^5^ In our study, all patients underwent upper endoscopy and histological evaluation; those with organic pathologies were excluded. Patients with Hp infection were treated according to national guidelines, and only those with persistent symptoms after standard therapy (defined as at least 4 weeks of proton pump inhibitors, prokinetics, and, when required, low-dose antidepressants) were considered within the FD framework. Of note, none of the patients with EPI in our cohort were Hp positive, which further strengthens the association between EPI and refractory FD.

We concur that our results should be interpreted with caution, yet they highlight an underappreciated overlap between FD and EPI.^6^ We hope that our study stimulates larger, multicenter investigations that will refine the diagnostic pathways for dyspeptic patients. We thank the correspondent for underscoring the clinical importance of this topic and for contributing to a constructive academic dialogue.

Sincerely,

Fatih Kemik, MD

on Behalf of the Authors

## Data Availability

The data that support the findings of this study are available on request from the corresponding author.
